# Exploration of neural correlates of movement intention based on characterisation of temporal dependencies in electroencephalography

**DOI:** 10.1371/journal.pone.0193722

**Published:** 2018-03-06

**Authors:** Maitreyee Wairagkar, Yoshikatsu Hayashi, Slawomir J. Nasuto

**Affiliations:** Brain Embodiment Lab, Biomedical Engineering, School of Biological Sciences, University of Reading, Reading, United Kingdom; Tokai University, JAPAN

## Abstract

Brain computer interfaces (BCIs) provide a direct communication channel by using brain signals, enabling patients with motor impairments to interact with external devices. Motion intention detection is useful for intuitive movement-based BCI as movement is the fundamental mode of interaction with the environment. The aim of this paper is to investigate the temporal dynamics of brain processes using electroencephalography (EEG) to explore novel neural correlates of motion intention. We investigate the changes in temporal dependencies of the EEG by characterising the decay of autocorrelation during asynchronous voluntary finger tapping movement. The evolution of the autocorrelation function is characterised by its relaxation time, which is used as a robust marker for motion intention. We observed that there was reorganisation of temporal dependencies in EEG during motion intention. The autocorrelation decayed slower during movement intention and faster during the resting state. There was an increase in temporal dependence during movement intention. The relaxation time of the autocorrelation function showed significant (*p* < 0.05) discrimination between movement and resting state with the mean sensitivity of 78.37 ± 8.83%. The relaxation time provides movement related information that is complementary to the well-known event-related desynchronisation (ERD) by characterising the broad band EEG dynamics which is frequency independent in contrast to ERD. It can also detect motion intention on average 0.51s before the actual movement onset. We have thoroughly compared autocorrelation relaxation time features with ERD in four frequency bands. The relaxation time may therefore, complement the well-known features used in motion-based BCI leading to more robust and intuitive BCI solutions. The results obtained suggest that changes in autocorrelation decay may involve reorganisation of temporal dependencies of brain activity over longer duration during motion intention. This opens the possibilities of investigating further the temporal dynamics of fundamental neural processes underpinning motion intention.

## Introduction

Brain computer interfaces (BCIs) that use electroencephalography (EEG) are being increasingly used in research due to their non-invasive nature and their potential in therapeutic applications such as motor rehabilitation [1–4]. BCIs allow users to control computers, robots or other devices directly via their brain activity and hence, could potentially enable patients with spinal cord injuries or other motor disabilities to interact with devices by producing tailored mental activity. BCI is commonly driven by paradigms involving evoked activity, such as steady state visually evoked potentials (SSVEP) [5, 6], event-related potentials (ERP) [7], as well as motor-related paradigms e.g. motor imagery [8]. SSVEP and ERP employ visual and attention processes, and they always require an external stimulus to evoke a detectable response. On the other hand, neural correlates of movement enable intuitive control of BCIs by producing movement intention at will without requiring any external stimuli [9, 10]. Typically, changes in power at specific EEG frequency bands are used for detecting movement intention. However, this disregards the movement related information present in the rest of the EEG spectrum and in the time domain, as the EEG signal is essentially non-stationary. In this paper, we explore movement related information which is not reflected in the band power changes, by studying the evolution of temporal dependencies in the EEG during motion intention.

The detection of voluntary movement intention, execution and imagery from EEG is typically achieved by widely used neural correlates of movement namely event-related (de)synchronisation (ERD/S) and motor-related cortical potential (MRCP) [10–14]. ERD and ERS corresponding to attenuation and increase predominantly in *μ* and *β* power respectively [11], are commonly used for detecting movement intention and imagery [12]. Based on this, most features for detecting movement related tasks [9] are extracted from the spectral domain of the EEG. Power spectral density (PSD) and time-frequency analysis being by far the most common methods of evaluating ERD [13–15]. Even though ERD/S can detect motor imagery with high accuracy [16, 17], these spectral features have to be tuned to individuals as most responsive frequency bands are different for each individual. Often, different spatial filters or other optimization techniques are used to enhance the spectral features [17–20]. MRCP is a slow negative cortical potential observed in low frequencies [13] from approximately 2s prior to human voluntary movement [21]. MRCP has a very small amplitude (8-10*μV*) compared to spontaneous EEG activity (100*μV*) [13] which makes its detection difficult. A common method of detecting MRCP is to average several EEG trials of voluntary movements [13]. ERD/S and MRCP have been used to distinguish between different types of movements involving different joints [22–24]. It is also possible to determine the end of movement planning, execution and imagery from the increase in *β* power also known as *β* rebound [25] and increase in *γ* power [15].

In addition to movement execution information, movement prediction is important for hybrid rehabilitation systems combining BCI and robotic assistance as it can compensate for the delay between the detection of the motion intention and the onset of motion assistance. Enabling the coordination of motion assistance with a subject’s motion intention can generate the ownership of the movement [26, 27] and improve the efficiency of a BCI-driven robotic rehabilitation systems [28]. ERD and MRCP can also be used for voluntary movement prediction before its actual onset. ERD power spectral density features in the *μ* and *β* bands were shown to predict movement on average 0.62±0.25s before actual movement onset [13], while a narrow frequency band of 0.01-1Hz gave a good movement prediction from -0.5s using MRCPs for reaching movement [29, 30]. For a unilateral hand movement, ERD occurs 2s before movement onset in the contralateral hand area of the sensorimotor region and becomes bilaterally symmetrical just before movement execution. ERD is followed by contralateral ERS within 1s of movement offset. ERD is also observed contralaterally during motor imagery [11]. In contrast, early component of MRCP starts 2s before movement onset, bilaterally distributed around midline-central region. The late MRCP component has larger amplitude and is observed 400ms before movement onset over the contralateral central region [21]. Combination of ERD and MRCP features provides complementary neurophysiological information about movement and improve the detection rate of movement intention [14, 31].

Various signal processing and statistical analysis techniques are increasingly being explored to improve the movement intention detection accuracy and decrease the latency between movement intention and its detection [32–34], however, the underlying principles of ERD/S and MRCP remain the same. The ERD and MRCP can detect movement intention only in specific narrow frequency bands. ERD also requires tuning of the most discriminative frequency band for each participant. Also, ERD and MRCP, though successfully employed to detect movement intention, do not completely describe all aspects of motor command generation, dynamical properties of brain activities, and changes in temporal dependencies. These characteristics of brain activities focus only on a selected frequency band. Thus, we explored a complementary process that will enable us to detect motion intention from a wider frequency band and which can be used in conjunction to ERD and MRCP to obtain a deeper insight into EEG dynamics during voluntary movement.

The aim of this paper is to identify novel neural correlates of movement based on changes in the dynamics of EEG by characterising its temporal dependencies, such that they provide movement intention related information that is complementary to ERD and MRCP. The study reported in [35] identified a specific change in the EEG autocorrelation during movement, which could be used as a marker of movement intention, however there were no further studies investigating the reorganisation of the autocorrelation structure during movement. Extending this line of investigation, we computed the autocorrelation function of EEG signals recorded during asynchronous finger tapping to evaluate the effect of the voluntary movement on the EEG dynamics. The changes in relaxation time of autocorrelation were observed during movement intention, and were then used as a novel time domain characteristic to classify movement intention from single trials. In this paper, we detected the motion intention before actual movement onset from the relaxation time of autocorrelation, and found that the autocorrelation relaxation time increases before and during voluntary movement, i.e. autocorrelation decays slowly during this period compared to the resting state. Thus, we obtained a neural correlate of motion intention based on characterisation of the temporal dependencies in the EEG.

The paper is organised as follows: in the Methods section, we provide details of the EEG experimental paradigm, EEG pre-processing, procedure for characterising motion intention from single trials using ERD and the novel autocorrelation relaxation time and classification of movement intention. In the Results section, we present the relaxation times of EEG autocorrelation in four frequency bands and compare them with ERD in the corresponding bands. We compared their classification sensitivities, timing of movement intention detection and spatial locations of the most responsive channel. This is followed by the Discussion section and the Conclusion.

## Methods

### Ethics statement

Fourteen healthy participants (8 female, age 26 ± 4 years, 12 right handed) with no prior experience in EEG experiments and BCIs, normal or corrected to normal vision, no motor or communication impairing conditions and not receiving any medication for such conditions participated in the study. Ethical approval for the EEG experiment was obtained from the ethics committee of the School of Systems Engineering, University of Reading, UK. Participants were provided with an information sheet detailing the purpose of the study, procedure of the experiment and the nature of the data collected. Informed written consent was obtained from all the participants before beginning the experiment.

### Experimental paradigm

A simple self-paced, asynchronous index finger tapping task was chosen to study the brain activities related to the motor intention.

**Fig 1:**
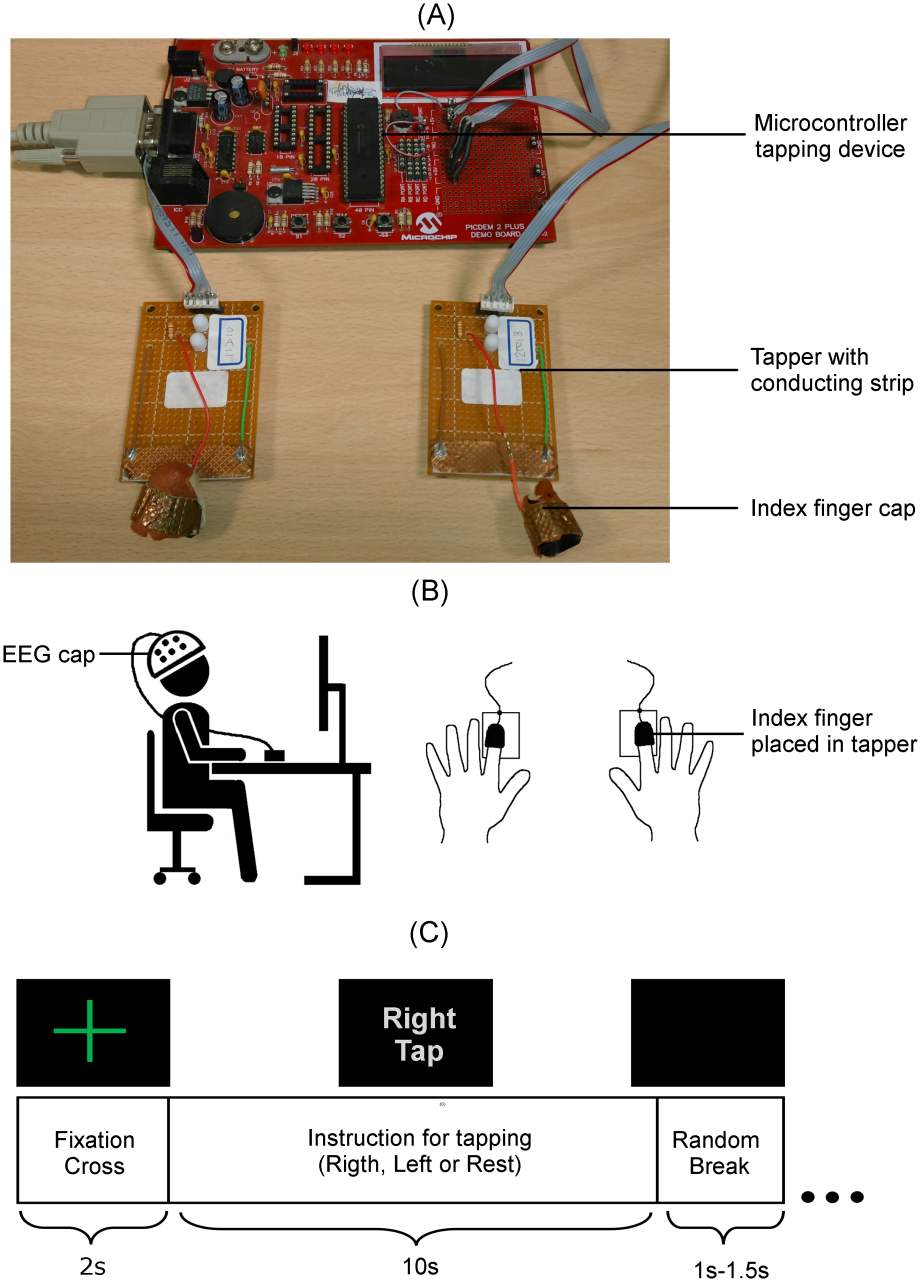
Experimental setup, devices and EEG trial structure. (A) Microcontroller tapping device to record right and left index finger taps, which were co-registered with the EEG to mark the tap onset. (B) Illustration of the EEG experimental setup and index fingers placed in the tapping device. (C) Structure of single EEG trial.

In order to record the onset of a finger tap, a bespoke microcontroller based tapping device was developed (Fig 1A) using an 8-bit Microchip PICDEM2 Plus demo board (Microchip Technology Inc., Arizona, USA). The tapping circuit consists of a finger cap covered with a conductive metal strip and a conductive plate placed on the tapping board and was connected to the pins on the I/O port of the Microchip board for peripheral devices. The participant was required to place the index fingers of both their hands in the corresponding finger caps of the tapping device as shown in Fig 1B, and perform the finger tap when instructed. The microcontroller sent a continuous 5V output to the tapper circuit and the analogue input from the right and left tapper was received on the input pins. This analogue input of 0-5V was converted to a digital signal in the range of 0 to 1023 by using the Microchip board’s inbuilt 10-bit analogue-to-digital converter. Thresholding was then applied to convert this signal into a binary stream (1 when there was no tap, and 0 when there was a finger tap) for each hand, which was then sent to the computer via a universal asynchronous receiver-transmitter (UART) to a serial port. This binary stream captured the onset and duration of each finger tap. These additional two channels of binary tapping signals, one for each hand were recorded at a sampling frequency of 1000Hz.

The experimental paradigm was developed in MATLAB Simulink R2014a (The MathWorks, Inc., Natick, Massachusetts, United States) using the BioSig toolbox [36]. The structure of a single trial is illustrated in Fig 1C. The experimental procedure was as follows:

1. Participants placed their index fingers in the tapping device as shown in Fig 1B.
2. A fixation cross was displayed for 2s on the screen.
3. This was followed by an instruction displayed in random order for a single right or left finger tap or the resting state.
4. Participants were given a 10s window to perform the instructed task at a random time of their choice and were instructed to avoid tapping immediately after the display of the instruction to avoid cue effects. During the resting state, participants stayed still without thinking of anything in particular.
5. Each trial was followed by a random break of 1-1.5s.

The EEG was recorded using a Deymed TruScan 32 EEG amplifier (Deymed Diagnostic s.r.o., Hronov, Czech Republic) and EASYCAP EEG cap with Ag/AgCl ring electrolyte gel based electrodes (EASYCAP GmbH, Herrsching, Germany) with 1.5mm touchproof safety sockets. Nineteen EEG channels according to the international 10-20 system were recorded using a referential montage with reference on FCz and ground on AFz. The cap was placed on the participant’s head such that Cz was located exactly on the central point between nasion and inion and right and left ear which was measured with a tape. The Deymed digital amplifier is battery operated to reduce artefacts and outside noise and has an inbuilt impedance monitoring system. The impedances of all the electrodes were kept below 7*k*Ω. The EEG was sampled at 1024Hz to obtain sufficiently high temporal resolution. No filtering was done on the EEG during recording. 40 trials per condition were recorded for each participant to obtain sufficient statistical power while minimising experiment time to avoid fatigue in the participants.

The EEG and finger tapping signals were recorded and co-registered together with the stimulus markers on the computer using the tools for brain-computer interaction (TOBI) SignalServer 2.0 which is a program for signal acquisition, co-registration and transmission that uses a standardised TOBI interface A protocol [37]. This co-registered signal was saved using the BioSig toolbox. This ensured that onset of the movement was accurately marked on the EEG time series. EEG data is available from http://dx.doi.org/10.17864/1947.117 [38].

### Pre-processing and artefact removal

The offline EEG analysis was performed to identify the neural correlates of voluntary movement intention. All EEG analysis and statistical analysis was done in MATLAB. EEG signals were filtered using a fourth order zero-phase non-causal Butterworth filter to avoid phase distortions in the filtered signal. The DC offset was removed by high-pass filtering with a 0.5Hz cut-off frequency. A notch filter at 50Hz was used to remove the power line noise. High frequency noise was eliminated by low-pass filtering with a cut-off frequency at 60Hz. Baseline noise caused by the drift in the EEG was removed by subtracting the mean of the signal, making it a zero mean, before artefacts removal and extraction of individual trials.

The EEG was visually inspected for large spikes and irregular artefacts [39], neither of which were observed in the data. Following this, artefacts removal was performed using independent component analysis (ICA) [40]. The independent components with artefacts were identified and eliminated manually using the EEGLAB toolbox for MATLAB [41], which uses an automated version of infomax ICA algorithm [42]. The reconstructed uncontaminated signal was visually inspected for any residual large artefacts and any undesirable trials were eliminated. EEGLAB was only used for artefacts removal using ICA.

All channels were re-referenced using a longitudinal bipolar montage, which enhances the signal by eliminating similarities in adjacent channels leading to magnification of the features associated with that region and suppression of the common noise between the channels [43]. Bipolar montage is a simple and fast spatial filter [43] commonly used for BCIs to reduce the number of channels [44–46], improve the signal-to-noise ratio by reducing the background noise in the EEG and significantly improve the classification results [47, 48]. Nine EEG channels surrounding the motor cortex (F3, Fz, F4, C3, Cz, C4, P3, Pz and P4) were used to obtain six bipolar channels, viz. F3-C3, Fz-Cz, F4-C4, C3-P3, Cz-Pz and C4-P4. Time locked trials of length 6s were obtained by extracting 3s before and 3s after the onset of the finger tap. These trials were divided into 1s sliding windows from time (t-1s) to t which were shifted by 100ms for further EEG analysis. Thus, each feature at time t was obtained on a window from (t-1s) to t. The baseline was removed from each trial to make it a zero mean.

### Characterising grand average and single trial ERD

To confirm that the recorded EEG data had captured the movement related information during finger tapping, the grand average ERD was estimated using event-related spectral perturbation [49] and the band power method [11].

Spectral perturbation of EEG was obtained via a short-time Fourier transform along the length of a 6s trial. To observe ERD, the average spectrogram of the resting state trials was subtracted from the average spectrogram of the movement trials. Data from all participants were visually inspected for the presence of ERD. The grand average spectrogram was obtained by averaging results across all the participants.

ERD was also identified using the band power method [11] by, firstly, band-pass filtering in a selected frequency band, and subsequently squaring and averaging the trials. The mean of corresponding samples in all trials was subtracted from each sample. For further smoothing and minimising the effects of spurious peaks or outliers on the band power, the mean of the upper and lower envelopes of data was used to compute ERD. This smoothing step was partly inspired by the techniques described in [50, 51] where they use amplitude envelope of the EEG to get the band power. The mean of 1.5s to 2s prior to movement onset was used as a baseline (*R*). This baseline was then subtracted from the mean of a 1s window (*A*) shifted by 100ms over the trial. ERD percentage was obtained for each window (*i*) using Eq (1). The grand average of ERD for six channels was obtained by averaging the corresponding ERDs of all the participants.

$$\begin{array}{c} ERD\left(i\right)=(\frac{A(i)-R}{R})\times 100 \end{array}$$ (1)

Single trial ERD was obtained as above, but on individual trials. The upper envelope of the squared EEG amplitude of each trial was used to get the baseline (*R*) and mean of each window (*A*). Eq (1) was used to get percent ERD for each trial.

### Characterising autocorrelation relaxation time of single trial EEG by fitting exponential curve

The autocorrelation function captures the changing EEG dynamics and shows how the temporal dependencies change over time during voluntary movement [35, 52]. The decay trend of the autocorrelation provides an insight into how the signal is related to its past. Hence, the autocorrelation decay of the EEG was studied during finger tapping to investigate the influence of motion intention on temporal dependencies by estimating the relaxation time of autocorrelation.

The autocorrelation of each trial was obtained to study the time development of the relaxation process of the brain activity before, during and after the finger tapping movement. The decay trend of autocorrelation was used as a measure of reorganisation of brain dynamics. Let *s* = *s*(0), *s*(1), *s*(2), …, *s*(*N*) be a discrete EEG signal with *N* samples. Then, the autocorrelation of *s* at a lag Δ is defined by Eq (2), where $\bar{s}$ represents the mean of signal *s*.

$$\begin{array}{c} R\left(\Delta \right)=\frac{\sum_{t=0}^{N-\Delta }\left(s\left(t\right)-\bar{s}\right)\left(s\left(t+\Delta \right)-\bar{s}\right)}{\sum_{t=0}^{N}{\left(s\left(t\right)-\bar{s}\right)}^{2}} \end{array}$$ (2)

At Δ = 0, the signal is perfectly correlated (*R*(0) = 1). At an infinite time lag, the signal components are completely uncorrelated (*R*(∞) = 0). How the signal becomes uncorrelated over time can be described by the trend of autocorrelation decay. We assumed that the relaxation process of the brain signals can be understood in terms of the models using the damped mass-spring system, the dynamics of which can be thought of in terms of a relaxation process and an oscillatory process and can be represented by the general form $Ae^{-\frac{t}{\tau }}cos\left(\omega t-\phi \right)$. Hence, we have obtained the first order approximation of the autocorrelation relaxation process by assuming an exponential decay model for the upper envelope of the autocorrelation function. By using this approximation, the autocorrelation decay is asymptotically equal to the exponential decay represented by Eq (3) over the length of a signal with *N* samples, where *C* > 0. Even if the autocorrelation decay is not exactly exponential, but follows a different trend, the exponential decay model may still quantify how fast or slow the autocorrelation decays.

$$\begin{array}{c} R\left(\Delta \right)∼Ce^{-\frac{t}{\tau }} \end{array}$$ (3)

**Fig 2:**
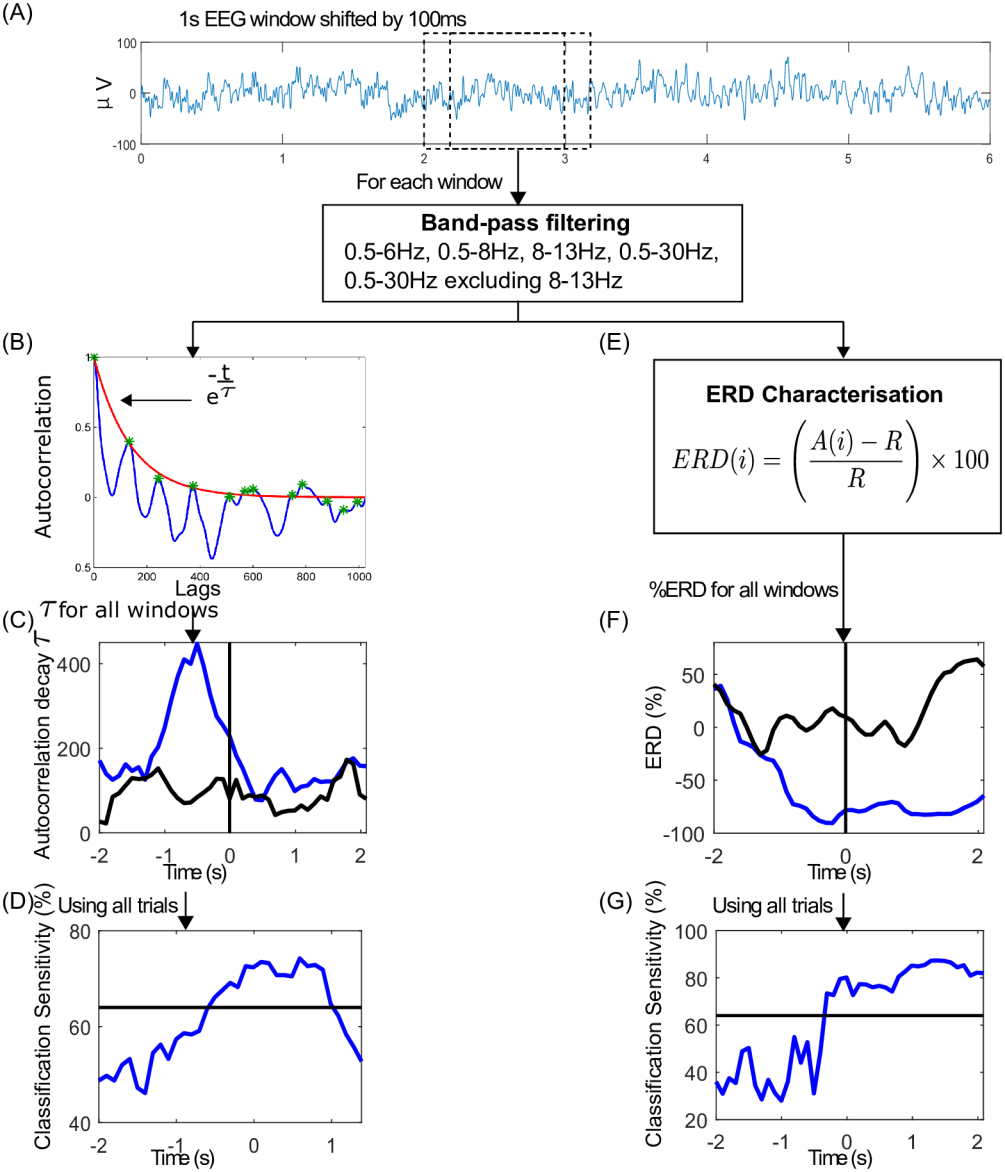
Autocorrelation relaxation time and ERD feature extraction and classification. (A) 6s EEG trial is divided into 1s windows shifted by 100ms and band-pass filtering is performed for the four different frequency bands. (B) Exponential curve fitting representing the autocorrelation relaxation process on each window. The relaxation time *τ* was extracted as a feature. (C) Plot of all *τ* values in a single trial for tap (blue) and resting state (black) (vertical line marks the onset of finger tap at 0s). (D) Classification sensitivities for tap vs. resting state along the length of trial for autocorrelation feature. The horizontal line indicates the classification threshold with statistical significance (*p* < 0.05). (E) Computation of ERD(%) for each window. (F) Plot of all ERD values in a single trial for tap (blue) and resting state (black) (vertical line marks the onset of finger tap at 0s). (G) Classification sensitivities along the length of trial for tap vs. resting state ERD.

Normalised autocorrelation was performed on a single trial basis using Eq (2). Each 6s EEG trial was divided into 1s windows shifted by 100ms. The EEG processing steps are shown in Fig 2.

Based on the approximation of the autocorrelation decay using the exponential function, the relaxation process of autocorrelation was obtained by fitting the exponential curve (Eq (3)) to the autocorrelation. The decay constant *τ* corresponds to the relaxation time and is used as a feature for movement intention detection (see Fig 2B). The exponential curve given by Eq (3) was fitted to the local maxima of half of the total positive lags of the autocorrelation of each 1s EEG windows. Here, *C* = 1, because the autocorrelation was normalized. How fast or slow the autocorrelation decays with respect to the movement intention was studied by observing the *τ* values over the period of the trial as shown in Fig 2C. Time progression of *τ* values during right and left finger tapping trials were compared with corresponding *τ* values of the resting state trials and were used for classification of movement (see Fig 2D).

### Comparison of autocorrelation relaxation time with ERD in multiple frequency bands

We have represented movement intention with two features viz. autocorrelation relaxation time and ERD. To examine if the changes in the relaxation time of autocorrelation during movement were more prominent in specific frequency bands, single trial autocorrelation analysis was performed independently on three non-overlapping frequency bands: 0.5-8Hz (lower frequencies), 8-13Hz (middle frequencies, *μ* band), 13-30Hz (higher frequencies), along with the wide frequency band of 0.5-30Hz covering the entire spectrum.

These broader frequency bands were chosen to analyse the changes occurring in the wider frequencies during movement, and because the most responsive frequency band for ERD differs from individual to individual [11, 53]. Wider frequency bands contain all the information from the individual specific responsive frequency bands while mitigating the inter-participant variability and increase robustness of the results. This gave a fair mode of comparison of the two distinct features representing movement intention. The new single trial autocorrelation relaxation time feature was compared with the corresponding single trial ERD feature obtained in the four frequency bands described above.

#### Classification of movement intention

Classification was done using a binary linear discriminant analysis (LDA) classifier for autocorrelation relaxation time and ERD features for right tap vs. resting state and left tap vs. resting state independently for each participant. LDA analysis was done on each of the six bipolar channels independently. A separate LDA classifier was trained for each sliding window with all the corresponding features from windows in all the trials. Thus, for each LDA, there were 40 data samples with a single feature for each class. Sensitivity, also known as the true positive rate, was computed by dividing the number of trials correctly classified as tapping trials by the total number of tapping trials. A 10x10 fold cross-validation scheme was used to obtain the classification sensitivities at the time points given by the 1s sliding windows with 80% of the data from corresponding windows from all the trials used as training data and 20% as testing data. The sensitivities of 6s trials for each of the six channels were plotted for all the participants as illustrated in Fig 2D and 2G. The 95% confidence level for binary classification (tap or rest) was obtained from the binomial distribution with *n* = number of EEG trials available and *p* = 0.05.

## Results

In this section, firstly, we report the movement related changes occurring in the ERD and relaxation time, and then compare both these features in terms of their classification sensitivity, timing of movement intention identification and spacial location of the most responsive feature. From these three measures, we have observed that relaxation time indeed provides information about movement intention that is distinct from ERD.

### Grand average ERD

ERD was used to validate the recorded EEG data for presence of movement related features.

#### Event-related spectral perturbation

**Fig 3:**
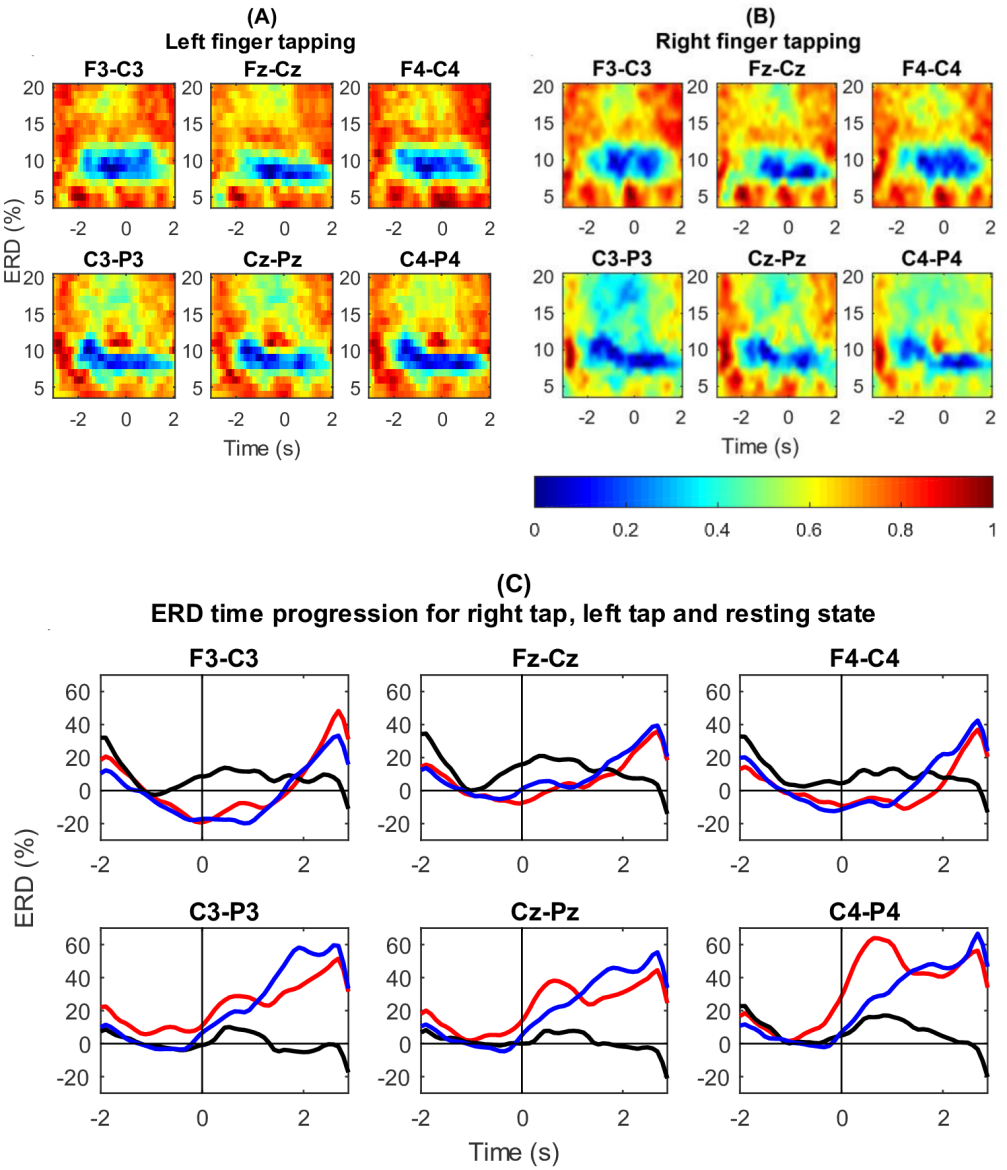
Grand average spectrograms and ERD time progression for six bipolar channels for all the participants. (A) Left finger tapping grand average spectrogram in six bipolar channels (the onset of finger tap is at 0s). ERD is clearly seen around 10Hz. (B) Right finger tapping grand average spectrogram in six bipolar channels (the onset of finger tap is at 0s). ERD is clearly seen around 10Hz. (C) Grand average ERD time progression for right finger tapping (red), left finger tapping (blue) and resting state (black). Vertical line shows the onset of finger tap at 0s. ERD is seen in right and left hand finger tapping.

ERD was observed from the grand average EEG spectrograms over all participants corresponding to left tap and right tap as shown in Fig 3A and 3B. A relative decrease in *μ* power is observed around 10Hz in all six channels.

#### Time progression of ERD

Time progression of the grand average ERD in the *μ* band of all the participants is plotted in Fig 3C. The plots for right (red) and left (blue) finger tapping drop below zero indicating the occurrence of ERD around motion onset (0s). The black plot represents resting state trials where no ERD is observed. ERD is seen more prominently in channels F3-C3, Fz-Cz and F4-C4. Channels C3-P3, Cz-Pz and C4-P4 show ERS after the onset of the movement.

Thus, ERD was present in EEG, validating the EEG for motion intention.

### Change in autocorrelation relaxation time related to motion intention

**Fig 4:**
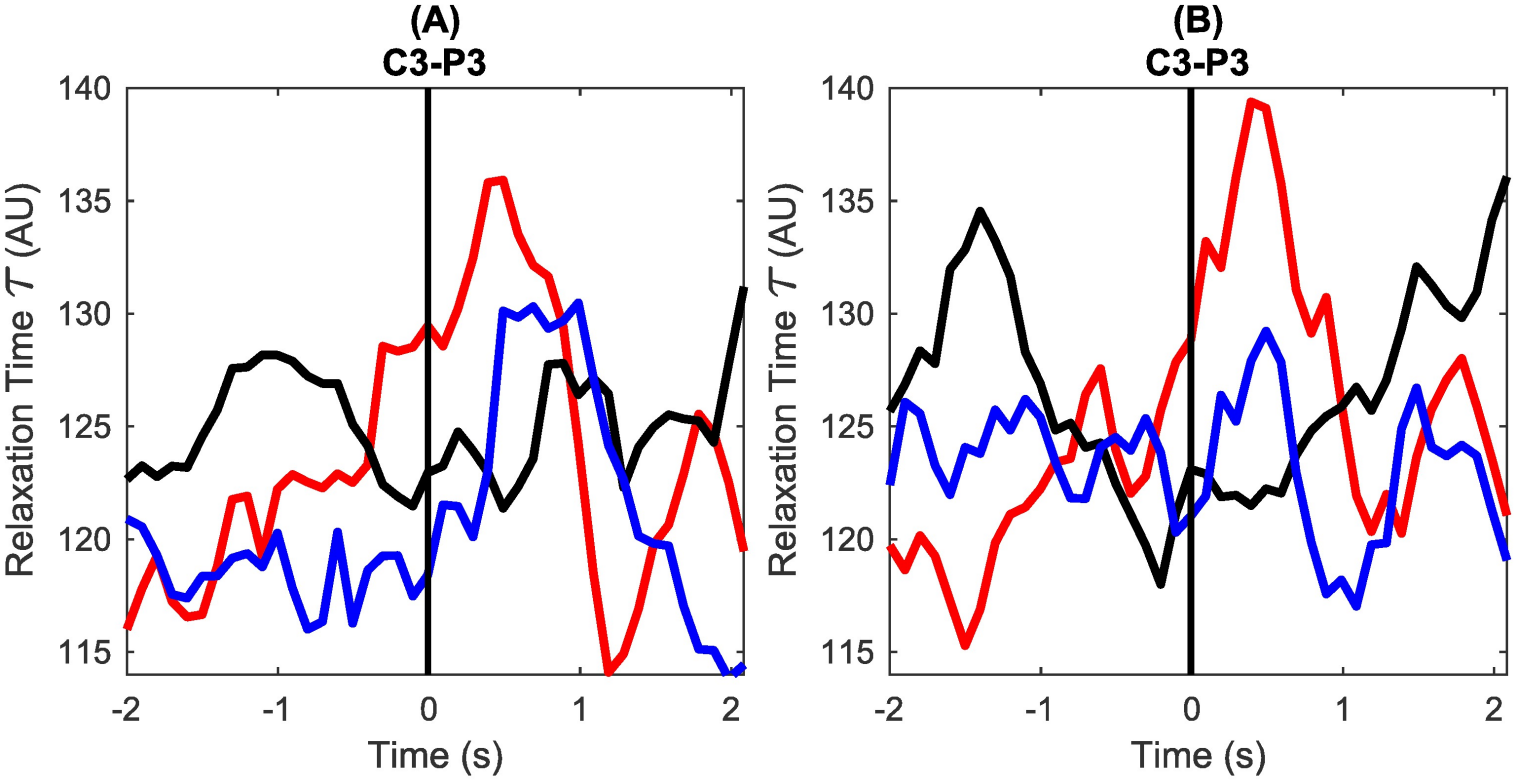
Grand average autocorrelation relaxation time (*τ*) for right finger tap, left finger tap and resting state for all participants. (A) Plot of grand average *τ* in channels C3-P3 for right finger tapping (red), left finger tapping (blue) and resting state (black), in broad frequency band 0.5-30Hz. The vertical line marks the onset of finger tap at 0s. (B) Plot of grand average *τ* in channel C4-P4 for right finger tapping (red), left finger tapping (blue) and resting state (black) in 0.5-30Hz band. Clear increase is seen around the movement in right and left hand tapping conditions.

It was observed that there was a change in the decay of autocorrelation around the movement onset. The relaxation time *τ* increased starting prior to the onset of movement and persisted during the movement duration. This increase in *τ* around the onset of movement was observed in most single trials as shown in the example in Fig 2C. The *τ* values for resting state trials did not show any such increase. The grand average *τ* for all the participants for right tap (red), left tap (blue) and resting state (black) for two channels is shown in Fig 4. Since most reactive channels were different in different participants, the aggregate build-up of *τ* was observed clearly only in C3-P3 and C4-P4 in the grand average. Relaxation time *τ* is large when the exponential curve decays slowly and small when the exponential curve decays fast. Thus, build up of *τ* prior to the onset of movement facilitates the prediction of movement before its actual occurrence.

The quality of fit of the exponential curve was assessed using the *R*^2^ value of the fit. The average *R*^2^ values for 0.5-8Hz, 8-13Hz, 13-30Hz and 0.5-30Hz were 0.89, 0.90 0.82 and 0.87 respectively, while the average range for *R*^2^ values for all the frequency bands was 0.3 to 0.99. There was no difference in the *R*^2^ values for exponential fitting in tap vs. no tap conditions. The *τ* features around the onset of the movement showed statistically significant differences between tap vs. rest conditions (*p* < 0.05, two sample t-test) for all the participants. Relaxation time of autocorrelation is hence, a robust way of characterising the changing dynamics of the brain activity during motion intention on a single trial basis.

### Comparison of autocorrelation relaxation time and ERD in different frequency bands

The three measures obtained from LDA classifier viz. classification sensitivities, estimated timings of motion intention detection and spatial locations of most responsive bipolar channels were compared. The sensitivities were obtained from 1s windows extracted from time (t-1s) to t shifted by 100ms.

#### Classification sensitivities of single trial autocorrelation relaxation time and ERD

**Fig 5:**
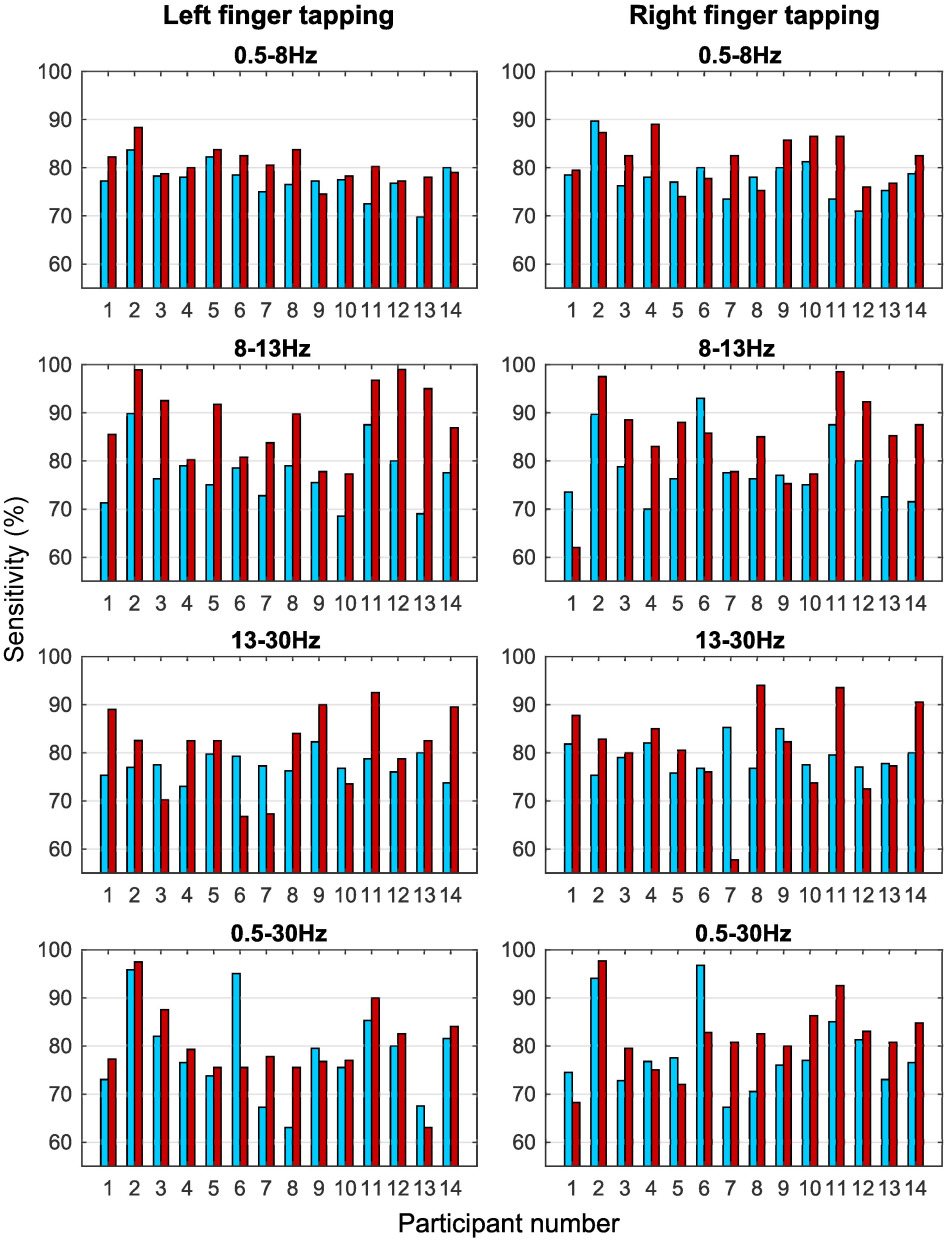
The peak classification sensitivities for autocorrelation relaxation time and ERD. The comparison bar graphs of right tap vs. rest and left tap vs. rest peak classification sensitivities (%) for all the participants in the four different frequency bands for autocorrelation relaxation time (blue) and ERD (red) features. These sensitivities are obtained from one of the six bipolar channels that shows the highest classification sensitivity in each case.

The maximum sensitivities for all the participants for right/left tap vs. rest were statistically significant (*p* < 0.05) for autocorrelation relaxation time. Classification sensitivities for all the participants in different frequency bands varied with a small standard deviation (SD). The best results were obtained in the wide frequency band of 0.5-30Hz for autocorrelation analysis. A peak sensitivity of 96.75% was achieved for participant number 6. The comparison of peak classification sensitivities for autocorrelation features for all the participants in all the frequency bands with the corresponding peak ERD classification sensitivities is shown in Fig 5.

**Fig 6:**
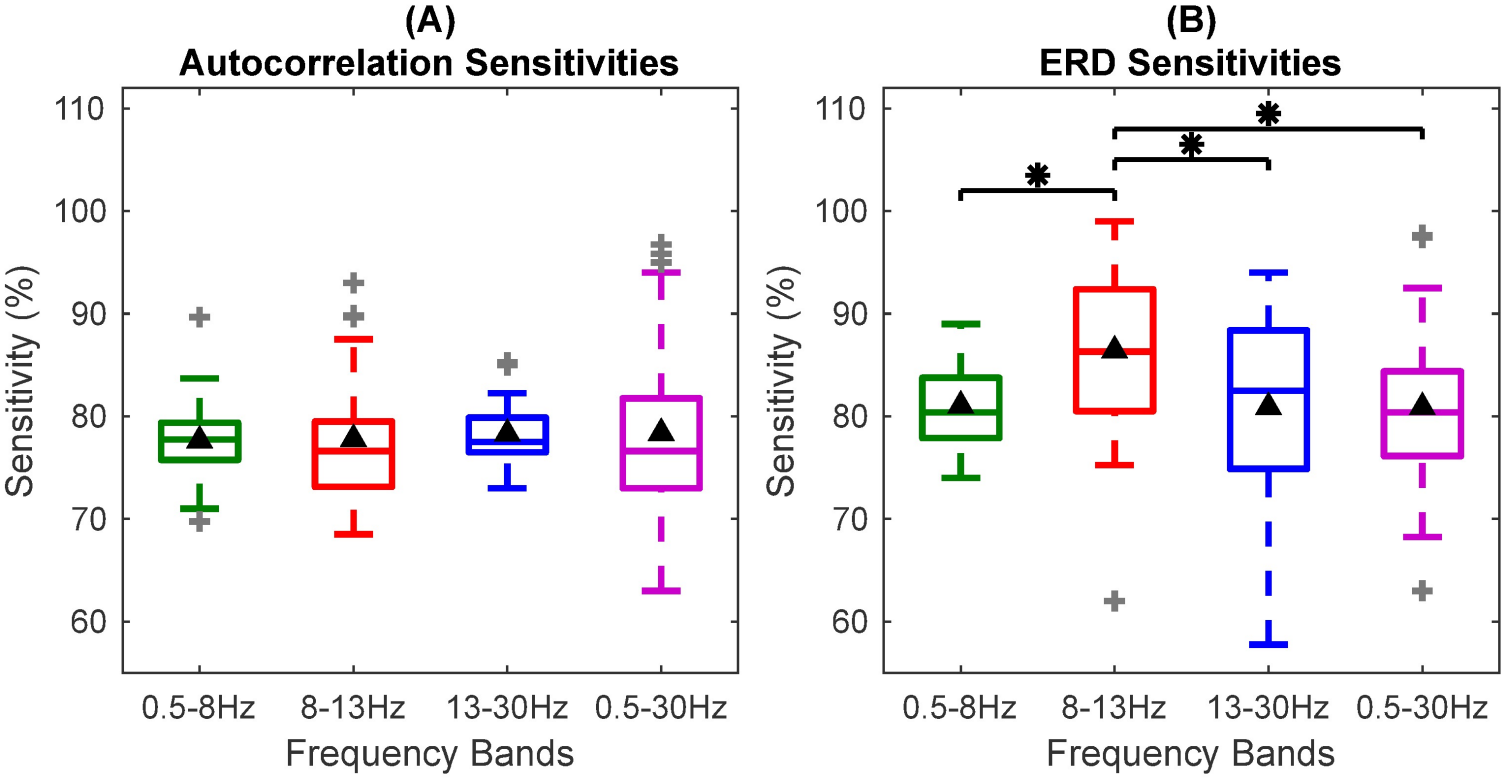
Box plot for comparison between classification sensitivities of autocorrelation relaxation time and ERD in four frequency bands. (A) The box plot for classification sensitivities of autocorrelation features in the four different frequency bands. The horizontal line in the box shows the median and black triangle shows the mean sensitivity. No significant difference is observed in sensitivities of different frequency bands. (B) Box plot of classification sensitivities of ERD features in four frequency bands. The sensitivities in the *μ* band are significantly higher *p* < 0.05 than the sensitivities in the rest of the bands indicated by asterisks. These sensitivities are obtained from one of the six bipolar channels that shows the highest classification sensitivity in each case.

Fig 6 shows the box plots for the classification results. It is observed from Figs 5 and 6 that the sensitivities of autocorrelation and ERD are comparable and close to each other with some participants showing higher sensitivities for autocorrelation than ERD in different frequency bands. Mean sensitivities in all the frequency bands are statistically significant (*p* < 0.05) for autocorrelation relaxation time and ERD features, although some participants do not show significant sensitivities for ERD features. Best autocorrelation sensitivities were obtained for the wide frequency band of 0.5-30Hz. Also, the SD of autocorrelation features is smaller than ERD in the 0.5-8Hz, 8-13Hz and 13-30Hz bands. These results show that the ERD performance varied in different frequency bands while the performance of autocorrelation features was consistent over different frequency bands.

Statistical tests on sensitivities of different frequency bands for all participants were performed using the non-parametric repeated measures Wilcoxon signed-rank test. Correction for six multiple comparisons between four frequency bands was done using the Holm-Bonferroni method [54] at a significance level of 0.05. The Holm-Bonferroni method was chosen because it is uniformly more powerful and less conservative than Bonferroni correction for multiple comparisons. Sensitivities of autocorrelation features were not significantly different in the four frequency bands. This shows that classification sensitivities for autocorrelation are consistent and independent of the frequency bands. In contrast, ERD sensitivities of different frequency bands were significantly different from the 8-13Hz (*μ*) band (*p* < 0.05). ERD showed higher sensitivities in the *μ* band. Significant differences for ERD in different bands are shown in Fig 6B.

Thus, autocorrelation relaxation time proved to be a robust frequency independent feature associated with motion intention. The classification sensitivities for all the four bands together for autocorrelation and ERD features were significantly different (*p* < 10^−7^, Wilcoxon signed rank test). This demonstrates that autocorrelation relaxation time and ERD represent different information about movement intention.

#### Estimating timing of motion intention detection from autocorrelation relaxation time and ERD

The sensitivities of the relaxation time feature crossed the significance threshold (obtained using binomial distribution, *p* < 0.05) around the onset of the motion. In most of the participants, the sensitivities crossed the threshold before movement onset and then dropped back after the completion of the movement. This suggested that statistically significant classification was possible even before the actual onset of the movement for some trials. This opened up the possibility for movement prediction using autocorrelation relaxation time. The time at which the classification sensitivity crossed the threshold was noted for each participant.

**Fig 7:**
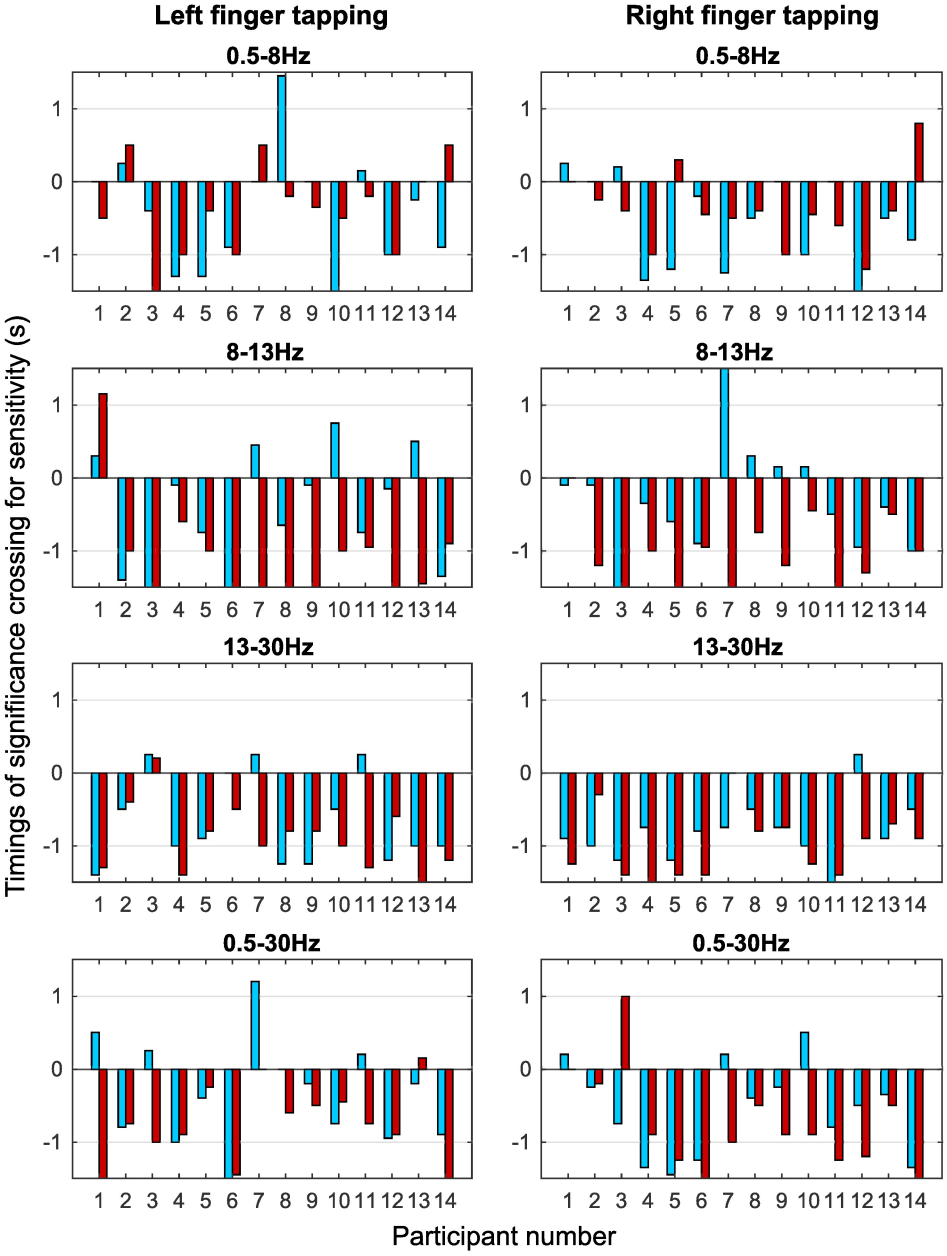
Timings for movement intention detection for autocorrelation relaxation time and ERD. The bar graphs show the time at which the classification sensitivity crossed the significance threshold indicating the classification between tap and resting state is above the chance level in four frequency bands for autocorrelation relaxation (blue) and ERD (red) features. Autocorrelation features as well as ERD features show negative values for time of movement intention detection indicting the prediction of movement before its onset. The timings are obtained from one of the six bipolar channels that shows the highest classification sensitivity in each case.

Fig 7 shows the comparison bar graphs for timings of the statistical crossing of the classification sensitivity for single trial autocorrelation and ERD features. It is observed from Fig 7 that prediction of movement is possible for most of the participants using autocorrelation analysis in all the frequency bands. In 0.5-8Hz and 0.5-30Hz bands for right and left hand tapping, many participants show movement intention detection as early as 1.5s to 0.5s prior to the onset of movement using autocorrelation relaxation time. ERD analysis shows earlier detection of movement intention in the 8-13Hz band as compared to the other bands as expected, whereas timing of movement intention detection was not frequency band dependent in autocorrelation analysis.

#### Spatial locations of most responsive bipolar channels for autocorrelation relaxation time and ERD

**Fig 8:**
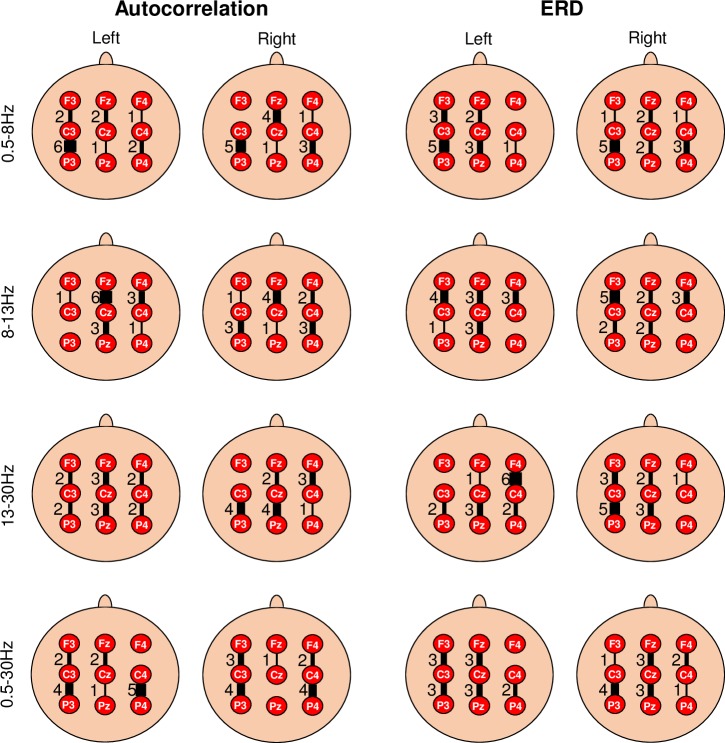
Spatial locations depicting most responsive channels for autocorrelation relaxation time and ERD. Spatial distribution of most responsive channel for autocorrelation relaxation time and ERD is shown for right tap and left tap in four different frequency bands. The width of the channel is directly proportional to the number of participants having that channel as most responsive channel showing maximum classification sensitivity. Number of participants having that channel as most responsive channel is also shown beside the channel location.

The bipolar channel that showed maximum classification sensitivity for each participant in each frequency band for autocorrelation relaxation and ERD feature was selected manually. Fig 8 shows the spatial locations of the most responsive channels for the classification of autocorrelation and ERD features. The sub-figures in Fig 8 depict the most responsive channel in different frequency bands. The width of a particular channel is directly proportional to the number of participants having that channel as the most responsive channel for classification. This is quantified by showing the number of participants having that channel as the most responsive channel beside the channel location.

The spatial locations of autocorrelation and ERD were different. Autocorrelation and ERD features have different most responsive channels suggesting that the spatial origin of the information about movement obtained from autocorrelation is different from the information obtained from ERD. The most responsive channels for ERD were obtained on the contralateral side, especially in the *μ* band (8-13Hz) and *β* band (13-30Hz). While typical ERD shows lateralisation in most cases [55], no lateralisation was observed in autocorrelation features. The most responsive channels for autocorrelation decay features differed from participant to participant. Autocorrelation did not show any distinct spatial pattern across different frequency bands, however, for a given frequency band, most participants had same most discriminative channel for both right and left hand movement.

Thus, our study indicates that 1) autocorrelation relaxation time increases during movement intention; 2) the classification sensitivities, timings of motion intention detection and spatial locations of most responsive channels are different for the autocorrelation feature and ERD; 3) changes in autocorrelation relaxation time are independent of frequency band; 4) autocorrelation provides different and complementary movement intention related information to ERD.

## Discussion

The aims of this paper are 1) to investigate reorganisation of the temporal dependencies in EEG in relation to motion intention and hence to put forward a novel neural correlate of voluntary movement preparation; 2) to determine if this new correlate provides complementary information about motion intention to ERD. This was achieved by studying the progression of changes in the EEG autocorrelation structure of single trials with asynchronous finger tapping. The change in the decay of autocorrelation as a function of lag indicates changing temporal dependencies in the signal. We assessed the reorganisation of these dependencies using an exponential function. The trend of autocorrelation decay was characterised by relaxation time, *τ*, which was computed by fitting a decaying exponential curve to the autocorrelation. To confirm that the autocorrelation relaxation time provides complementary information to ERD, the two single trial features were compared based on their classification sensitivities, timings and spatial locations.

The autocorrelation decayed fast during the resting state and slowly during motion intention and execution which suggests reorganisation of temporal dependencies prior to motion execution. When there is no movement, the EEG autocorrelation decayed fast indicating fluctuating recruitment of neural populations over time. Slow decaying autocorrelation may occur when EEG becomes self-similar or periodic, characterised by the presence of temporal dependencies over longer periods. The increase in relaxation time was observed even in single trials as shown in the example in Fig 2C and hence, it can be used as yet another feature to detect motion intention. Although the mean *R*^2^ value of exponential decay fit was high, it varied over a wide range of 0.3 to 0.99. Thus, there were still some trials and windows where the exponential decay was not a good fit, and perhaps these could be modelled better with a different decay trend like power-law.

Comparison of autocorrelation with ERD was performed because ERD is a well established phenomenon occurring during voluntary movement. However, ERD is highly frequency dependent and is only defined when a frequency band and a baseline are given [11]. ERD features in the *μ* band were significantly different from other frequency bands (*p* < 0.05) which is also commonly reported in the literature [10, 46, 56]. Significant presence of ERD was also observed in the 0.5-8Hz band which is not usually associated with movement ERD. However, ERD is not specific to movement and desynchronisation has been observed in a number of cognitive activities. Thus, presence of ERD in this frequency band could be due to the combination of factors such as influence of ERP components [57], desynchronisation occurring due to any task or attentional process [58], information encoding and memory [58, 59] or emotional intelligence [60]. Autocorrelation relaxation time on the other hand, was not frequency dependent and all the bands showed similar classification sensitivities which were tested using multiple comparisons with the Wilcoxon signed-rank test, with mean sensitivities in the narrow range of 77.11% to 79.24%, whereas, the mean ERD sensitivities varied over a wider range of 79.92% to 88.27% (see Fig 6). Autocorrelation analysis on broad band signal can identify movement intention without compromising the performance, unlike frequency specific ERD. Hence, we can conclude that some processes in the brain are represented by autocorrelation decay but not by ERD which fails to detect small changes in temporal dependencies. The wide band of 0.5-30Hz showed better overall sensitivities (though not significantly better) indicating that identification of this change in temporal dependencies became easier with a wide frequency range. During motion intention, there might be a global reorganisation of these temporal dependencies in a wider spectrum of brain activity. Thus, autocorrelation helps in gaining additional understanding of neural processes and opens a new avenue for further investigation of voluntary movement intention.

The autocorrelation relaxation decay can be used independently to detect motion intention with classification sensitivities comparable to the results reported in the literature using traditional MRCP and ERD, for example, 74.5 ± 13.8% in [14], 76% in [29, 30], 75% in [22], 79±11% and 68±10% in [32]. All these methods are based on features extracted from different channels independently and show similar results. To improve these classification accuracies and obtain holistic understanding of the movement generation process, alternative approaches like connectivity analysis between different regions [61] may help in conjunction with the autocorrelation relaxation time, ERD and MRCP.

Autocorrelation analysis is capable of identifying movement intention as early as -1.5s to -0.5s in many participants (see Fig 7), particularly in the 0.5-8Hz, 13-30Hz and 0.5-30Hz bands which can be used in future work to predict movement. This is comparable to the timings reported by [13] (-0.62 ± 0.25s) using *μ* and *β* powers and -0.5s [29, 30] using MRCP. There was no correlation between the motion intention onset time estimates obtained using autocorrelation relaxation and ERD features as seen from the bar graphs in Fig 7, which again suggests that both characteristics represent independent processes. This is further corroborated by the prominent spatial locations for the two features which were different in all the cases, with autocorrelation showing no lateralisation or spatial patterns as opposed to ERD, that was predominantly observed contralaterally in the *μ* and *β* bands as seen in Fig 8. Spatial locations of autocorrelation features were the same for right and left hand movement in many participants for a given frequency band. This may indicate that the change in autocorrelation decay purely characterises a fundamental correlate for voluntary movement intention and might not reflect on the handedness of the movement. Several cortical areas are involved in movement generation making it difficult to identify single specific spatial location [61]. Thus, autocorrelation decay features and ERD features have different spatial origins and represent two different movement related processes.

Even though the autocorrelation decay features are independent of frequency bands, they are not robust across different spatial locations. The spatial responsiveness of these features differ from individual to individual requiring manual selection of the most responsive channel for the movement intention detection. Further investigation is required to understand the spatial localisation of autocorrelation decay features in detail. This will then enable automated selection of characteristic spatial locations of autocorrelation features in the future.

The autocorrelation relaxation time is also complementary to small amplitude, low-frequency MRCP, because MRCPs are only observed clearly in lower frequencies [13], as opposed to the wide band prevalence of the autocorrelation decay. This was also shown in our previous study detailed in [62]. Thus, changes in relaxation time of autocorrelation represents a movement related process that is different from ERD and MRCP and possibly generated through a different neural mechanism, providing complementary information about movement intention related EEG temporal dynamics. Using autocorrelation in conjunction with ERD and MRCP correlates might lead to improved detection of motion intention. This will also help in assessing the complementarity of these features.

The autocorrelation analysis in our study was done on a single trial basis by characterising movement intention every 100ms from sliding windows of 1s. The analysis was designed in such a way that it could be easily adapted for implementation in an online BCI for movement intention detection in the future by using any online artefacts removal technique such as [63]. In this study, we have used non-causal zero-phase filters for pre-processing the data, however, these non-causal filters cannot be used in an online BCI implementation. Hence, from this study, we do not know the effects of causal filtering on the autocorrelation-based features.

Our analysis shows that the phenomenon of an increase in the autocorrelation relaxation time is observed during voluntary movement intention, yet further research is necessary to investigate if it occurs only during voluntary movement generation reflecting the motor tasks or whether it can be observed during other tasks as well. The fact that the relaxation time increases prior to the onset of movement as seen from the prediction timings that vary among different participants, suggests that in this case, it is more likely to reflect the change in EEG dynamics during motor-related tasks rather than changes due to a general task. This is also supported by a recent study in [64] that uses our autocorrelation decay features to discriminate the speed of hand movement characterising yet another aspect of a voluntary movement.

The apparent discrepancy between information about movement preparation, contained in the power spectrum-based ERD and our characterisation of autocorrelation relaxation trend, suggests some form of non-stationarity of the underlying processes. According to the studies reported in [65, 66], changes were observed in temporal dependencies in the brain activity in the 3-40Hz frequency range with the most significant change in the *α* band during neurofeedback despite no changes in the power spectrum. Thus, they suggested that neuronal circuits were more capable of reorganising the temporal dynamics than the magnitudes of the brain activity. Our results also show similar reorganisation of the EEG time series, indicating movement intention related modulation of temporal dependencies independent of changes in the power spectrum. The study in [65] concentrated on long-range dependencies using detrended fluctuation analysis (DFA), a technique proposed for the characterisation of the non-stationary processes. Further studies have also reported long-range dependence in EEG [67–69]. This suggests an interesting possibility that, the processes we observed during motion preparation may be non-stationary and hence, in future work, it would be interesting to investigate the nature of long-range correlation structure during motion intention using other approaches [70], such as DFA [71].

## Conclusion

In this paper, we have studied the changes in temporal dependencies in EEG related to voluntary movement intention. We have shown that a novel neural correlate of motion intention can be obtained in single trials from the modulations of the EEG autocorrelation structure characterised by its relaxation time. Autocorrelation decayed slower during motion intention and execution as compared to a resting state. Thus, the EEG became more self-similar during movement intention and execution due to this reorganisation of its temporal dynamics. Movement intention was detected using autocorrelation relaxation time on average 0.51s before its actual onset with the mean sensitivity of 78.37 ± 8.83% in the broad frequency band.

Information obtained from the autocorrelation relaxation time is different from ERD. It captures characteristics of movement intention that are not represented by ERD. The autocorrelation relaxation time is independent of frequency bands and represents a global change in the temporal dynamics of EEG in a wider spectrum. Thus, change in autocorrelation of EEG complements conventional ERD and MRCP processes and can be used in conjunction with the latter for improved movement intention detection in online BCI. This can again help in confirming their complementarity.

In future work, it would be interesting to explore further the impact of motion intention on long range temporal dependencies in EEG. Also, the proposed single trial neural correlate will be tested with online BCI for motion intention detection. It will also be interesting to investigate during which types of tasks does the autocorrelation relaxation time increase or whether it is specific to voluntary movement.
